# Inborn errors of immunity with DNA repair disorders: at the interface of immune deficiency, immune dysregulation, and malignancy

**DOI:** 10.3389/fimmu.2026.1815028

**Published:** 2026-04-28

**Authors:** Aleksandra Szczawińska-Popłonyk

**Affiliations:** Department of Pediatric Pneumonology, Allergy and Clinical Immunology, Institute of Pediatrics, Poznan University of Medical Sciences, Poznań, Poland

**Keywords:** DNA repair, immune dysregulation, immunodeficiency, inborn errors of immunity, malignancy

## Abstract

DNA repair disorders are characterized by defective DNA damage response and DNA replication pathways due to pathogenic variants in multiple genes involved in DNA repair, such as base excision, nucleotide excision, mismatch, non-homologous end joining repair machineries, as well as the homologous recombination process. They comprise a group of inborn errors of immunity of variable severity from hypogammaglobulinemia to severe combined immunodeficiency, accompanied by immune dysregulation and increased risk of malignant transformation. Clinical conditions present with a spectrum of syndromic features, including dysmorphism, microcephaly, neurodevelopmental delay, short stature, cutaneous lesions, and skeletal malformations. This review is aimed at defining the molecular underpinnings of DNA repair disorders, with special emphasis on clinical aspects and immune deficiency, immune dysregulation, and carcinogenesis. With the ever-increasing progress in immunogenetics and understanding the molecular pathology of DNA instability syndromes, due to the rarity and complexity of phenotype–genotype relationships, the diagnosis in affected patients remains challenging for clinicians. Increased awareness and vigilance in diagnosing and implementing the therapy are therefore required in individuals affected with notorious and multifaceted inborn errors of immunity and DNA repair disorders.

## 1 Introduction

**Figure 1:**
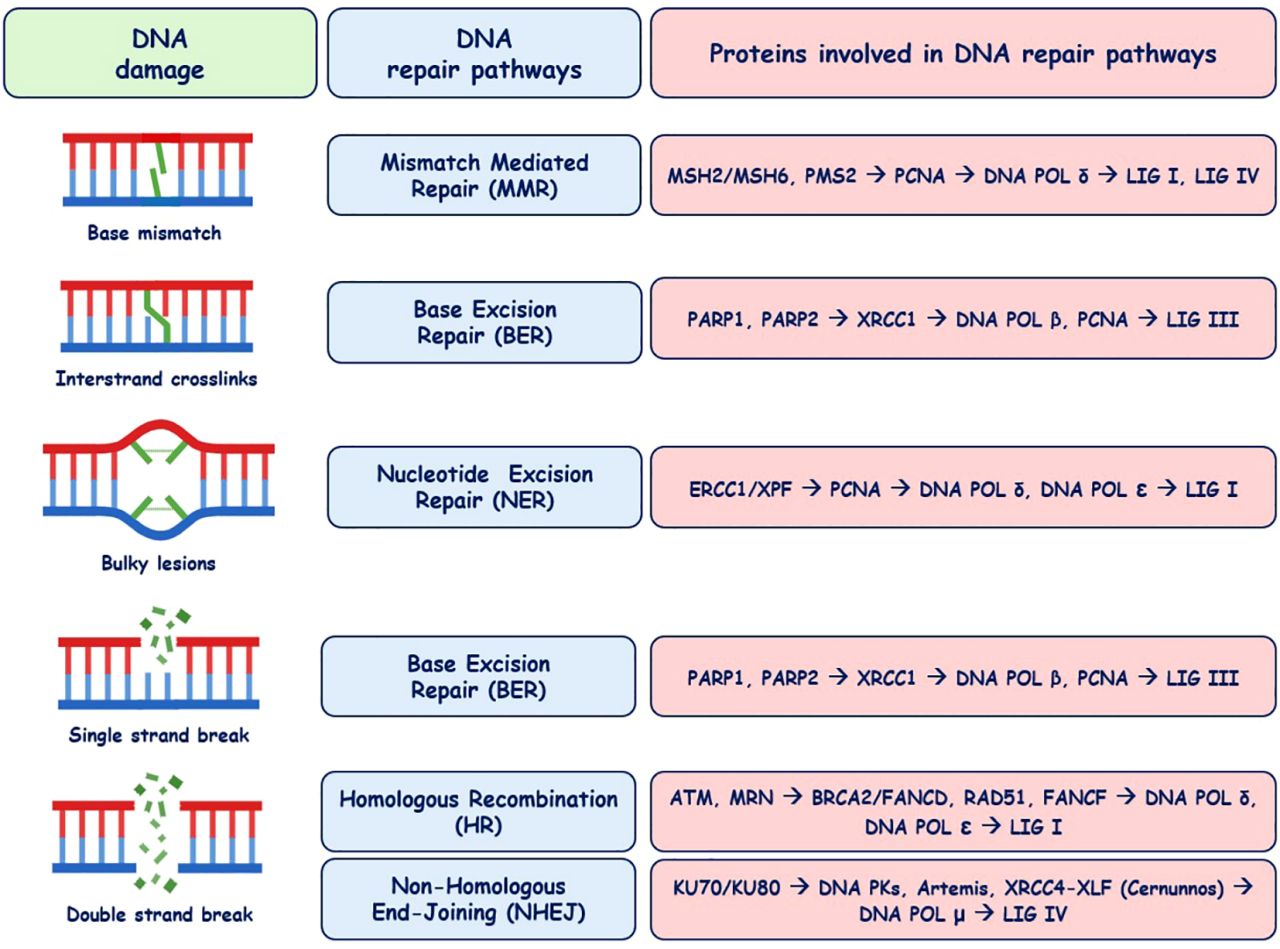
DNA damage and repair pathways with relevant proteins [according to (3) and (4)].

DNA repair disorders are a group of genetically and phenotypically heterogeneous clinical conditions characterized by developmental anomalies of various organs and systems, including dysmorphism, short stature, microcephaly, neurological deficiencies, accelerated aging, and increased susceptibility to malignant transformation (1). The genetic background for these disorders are pathogenic variants in genes encoding proteins that play a key role in DNA replication and DNA damage response, which result in genomic instability (2). Multiplicity of endogenous factors and external exposures can lead to the DNA lesions stalling the replication and transcription machinery, causing mutagenic, stress, or cell death responses (3). Intrinsic genotoxic factors include spontaneous hydrolysis, replication errors, replicative stress, and reactions with endogenous chemicals, such as reactive oxygen species (ROS) (3). Extrinsic genotoxic agents, which may result in mutations and genomic instability, are ionizing and UV radiation and chemicals, such as alkylating agents, aromatic amines, and cross-linking agents, or environmental factors, including heat, cold, and hypoxia (4). Maintaining genomic homeostasis and integrity requires cell cycle checkpoint controlling pathways, the DNA damage response (DDR), and DNA repair mechanisms. The endogenous complex system monitoring the genome integrity and sensing of DNA damage is involved in the recruitment of lesion-specific repair pathways. The nexus of proteins sensing DNA lesions, such as Fanconi anemia group D2 (FANCD2), xeroderma pigmentosum group C (XPC), H2A histone variant X (γH2AX), ataxia–telangiectasia mutated (ATM) kinase, DNA-dependent protein kinase catalytic subunit (DNA-PKcs), and MRE11–RAD50–NBS1 (MRN) complex, regulate and activate the repair cascades (5). These processes comprise DNA mismatch repair (MMR), base excision repair (BER), nucleotide excision repair (NER), single-strand break repair (SSBR), and double-strand break repair (DSBR). A network of reparation enzymes, such as nucleases, helicases, polymerases, and ligases, are engaged in DNA damage repair (1, 4, 6). DNA damage and repair pathways, including the involvement of reparatory proteins, are displayed in **Figure 1**.

Despite double-strand breaks being the most genotoxic and deleterious form of DNA damage—resulting in cellular apoptosis or extensive genomic instability with mutations and carcinogenesis—a precisely regulated DNA strand break process occurs physiologically in the rearrangement process of T cell receptor (TCR) and immunoglobulin B cell receptor (BCR) coding genes. The somatic recombination of variable (V), diversity (D), and joining (J) gene segments is initiated by lymphocyte-specific endonucleases RAG1/RAG2 through introducing DNA double-strand breaks (DSB). Consequently, reparation proteins are engaged to join RAG-processed DNA ends and form V(D)J rearrangements upstream of the constant region exons of immunoglobulin genes in B cells and TCR in T cells (7). The V(D)J recombination pathways provide the antigenic B cell and T cell receptor diversity, thereby ensuring the diversity of the antigenic repertoire and the specificity of adaptive immune responses.

Defects in DNA repair mechanisms give rise to a spectrum of inherited disorders characterized by distinct molecular backgrounds, chromosomal instability, and chromosomal breakage associated with heterogeneous syndromic phenotypes and susceptibility to develop malignancies (8, 9). Numerous chromosomal instability syndromes, beyond a wide array of clinical features, present with various developmental and maturational alterations in the B and T cell compartments, and hence they have been recognized and implicated in classifications of inborn errors of immunity (IEI) (10–12). In the current phenotypic and immunogenetic IEI stratification (10), majority of these disorders have been categorized as combined immunodeficiencies with DNA repair defects and subdivided into chromosomal instability syndromes with radiosensitivity (e.g., ataxia–telangiectasia (AT), Nijmegen breakage syndrome (NBS), Bloom syndrome (BS), ligase I deficiency, and NK cell deficiencies (e.g., Rothmund–Thomson syndrome), but they may also be found in the group of severe combined immunodeficiencies (SCID) (e.g., Cernunnos deficiency, ligase IV deficiency).

It is worth noting that the genetic heterogeneity governing aberrant DNA damage responses and chromosomal instabilities associated with immunodeficiency disorders overlaps with a wide spectrum of clinical conditions, such as developmental abnormalities and dysmorphism, as well as neurodevelopmental, muscular, skeletal, cutaneous, ocular, metabolic, and hormonal disturbances. The patient’s individual clinical and immunological features are thereby a common denominator of mutual genotype–phenotype correlations.

The following narrative non-systematic review was conducted to gather, summarize, and conclude the burden of the multifaceted presentations of DNA repair disorders and to highlight their systemic, immunogenetic, and malignant aspects in pediatrics. It was also aimed at increasing awareness of these disease entities among pediatricians to prevent diagnostic delay and facilitate the implementation of specialized treatment options.

The IEI disorders associated with DNA repair defects, including variants in relevant genes, and the leading clinical symptomatology with immunodeficiency are summarized in **Table 1**.

**Table 1:** Inborn errors of immunity associated with DNA repair disorders.

Inborn error of immunity	Gene	Phenotype	Immunodeficiency
Combined immunodeficiencies CID			
Ataxia–telangiectasia AT	*ATM*	Cerebellar ataxia, choreoathetosis, oculomotor apraxia, respiratory infections, telangiectasia, growth retardation, infertility, insulin resistance, granuloma, cafe-au-lait spots	Low TREC and KREC, restricted TCR and BCR repertoire, impaired lymphocyte neogenesis, defective generation of B and T memory cells, IgA deficiency, hyper or hypo IgM, low IgG and IgE
Nijmegen breakage syndrome NBS	*NBS1*	Microcephaly, neurodevelopmental delay, growth retardation, facial dysmorphism	Low TREC and KREC, restricted TCR and BCR repertoire, low IgG, IgA, and IgG subclasses, high IgM, T cell deficiency
Nijmegen breakage syndrome-like disorder NBS-LD	*RAD50*	Microcephaly, neurodevelopmental delay, growth retardation, facial dysmorphism	Progressive bone marrow failure, B and T cell deficiency
Bloom syndrome BS	*BLM*	Microcephaly, neurodevelopmental delay, growth retardation, facial dysmorphism, photosensitivity, erythema, insulin resistance	Impaired T and B cell maturation and memory cell generation, low IgG, IgM, and IgA
PMS2 (postmeiotic segregation increased 2) deficiency	*PMS2*	Cafe-au-lait macules, hypopigmented patches, brain malformations, cutaneous vascular anomalies	Low switched memory B cells and plasmablasts, decreased IgG, IgA, and antibody response, high IgM level
POLE1 (polymerase ϵ subunit 1) deficiency (IMAGe syndrome, FILS syndrome)	*POLE1*	FILS syndrome: Facial dysmorphism, livedo, short stature, recurrent infections IMAGe syndrome: Intrauterine growth restriction, metaphyseal dysplasia, adrenal insufficiency, genital anomalies, facial dysmorphism, recurrent infections	Low IgM and IgG2 subclass, lack of specific anti-polysaccharide antibodies, reduced numbers of B and NK cells, defective B cell maturation and generation of memory cells, impaired T cell proliferation
RNF168 (ring finger nuclear factor 168) E3 ligase deficiency (RIDDLE syndrome)	*RNF168*	Microcephaly, ataxia, short stature, facial dysmorphism, neurodegeneration, premature aging, learning difficulties, respiratory failure	Defective CSR pathway, reduced IgG and IgA, elevated IgM
DNA ligase I deficiency	*LIG1*	Short stature, failure to thrive, delayed sexual maturation, recurrent respiratory infections, chronic diarrhea, features of Omenn syndrome with exfoliative erythematous skin lesions, lymphadenopathy	Hypogammaglobulinemia with reduced IgG, IgM, and IgA levels, lymphopenia, impaired response to mitogens, increased proportion of γδ T cells, T-B-NK+ SCID
NSMCE3 (non-structural maintenance of chromosome (SMC) element 3) deficiency	*NSMCE3*	Severe interstitial pneumonia, respiratory failure, psychomotor disability, axial hypotonia, failure to thrive, hypoglycemia, eczema	T cell lymphopenia, impaired response to mitogens, normal to increased IgM levels, impaired antibody response to pneumococcal polysaccharide antigens
Immunodeficiency, centromeric instability, and facial anomalies (ICF) 1,2,3,4	*DNMT3B ZBTB24* *CDCA7* *HELLS*	Severe respiratory and gastrointestinal infections, failure to thrive, facial anomalies: hypertelorism, flat nasal bridge, epicanthus, low-set ears	Variable hypo- or agammaglobulinemia, impaired lymphogenesis, low B cell counts, deficiency of switched memory B cells, T cell lymphopenia – reduced RTE, naive, regulatory cells, increased follicular T helper cells
Severe combined immunodeficiencies T-B-NK+ SCID			
DNA ligase IV deficiency	*LIG4*	Severe respiratory, gastrointestinal, and urinary tract infections, microcephaly, facial dysmorphism: bird-like face, epicanthic folds, skeletal deformation, bone anomalies, failure to thrive, growth restriction, eczema, hypogonadism, psychomotor disability	Hypo- or agammaglobulinemia, low B cells, defective switched memory B cell generation, T cell lymphopenia including T-B-NK+SCID, low T reg counts, restricted TCR repertoire, impaired response to mitogens, neutropenia
Cernunnos/XLF (XRCC4-like factor) deficiency	*NHEJ1*	Severe respiratory infections, BCG-itis, failure to thrive, growth retardation, microcephaly, facial dysmorphism: bird-like face, developmental delay	Hypogammaglobulinemia, low B cells, T cell lymphopenia including T-B-NK+SCID, low TREC, impaired T cell response to mitogens, neutropenia
DNA PKcs (protein kinase catalytic subunit) deficiency	*PRKDC*	Recurrent lung infections, granulomatous skin lesions, neurodevelopmental delay, seizures, microcephaly, growth retardation, failure to thrive	Low T cell thymic output, loss of T cell naïvete, restricted TCR repertoire, defective T cell response to mitogens, T-B-NK+SCID/CID
Artemis/DCLRE1C (DNA cross-link repair 1C) deficiency	*DCLRE1C*	Recurrent respiratory and gastrointestinal infections, granulomatous skin lesions, cellulitis, warts, failure to thrive, growth restriction, inflammatory bowel disease	Reduced naive T cells, restricted T cell receptor repertoire, defective T cell proliferative response, low B cells, hyper IgM, low IgA or agammaglobulinemia and T-B-NK+ SCID
NK cell deficiencies			
MCM4 (minichromosome maintenance complex member 4) deficiency	*MCM4*	Growth retardation, failure to thrive, microcephaly, adrenal glucocorticoid failure, recurrent viral infections	Reduced numbers of CD56^dim^ and immaturity of CD56^bright^ NK cells
GINS1 (Go-Ichi-Ni-San complex subunit 1) deficiency	*GINS1*	Pre- and postnatal growth retardation, facial dysmorphism, hypothyroidism, recurrent viral infections	Neutropenia, NK cell CD56^dim^ and CD56^bright^ deficiency, high IgA, decreased IgM and IgG
GINS4 (Go-Ichi-Ni-San complex subunit 4) deficiency	*GINS4*	Prenatal growth restriction, postnatal short stature, criptorchidism, recurrent viral infections	Neutropenia, NK cell deficiency
MCM10 (minichromosome maintenance complex member 10) deficiency	*MCM10*	Fatal susceptibility to CMV, hemophagocytic lymphohistiocytosis	CD56^bright^ NK cell deficiency, decreased B and T effector and memory cells
Other immunodeficiencies			
POLE2 (polymerase ϵ subunit 2) deficiency	*POLE2*	Omphalitis, erythroderma in the newborn, facial dysmorphism, growth restriction, systemic BCG, lymphadenopathy, lung infections, autoimmune disorders: diabetes, hypothyroidism	Agammaglobulinemia, severe block in B cell development, T cell lymphopenia, absent TRECs, increased proportion of T effector memory cells, reduced NK cells, neutropenia
Rothmund–Thomson syndrome	*RECQL4*	Short stature, skeletal abnormalities, dental defects, juvenile cataracts, rash, poikiloderma, skin hyperpigmentation, ectodermal dysplasia, sparse hair, eyelashes and eyebrows, hypogonadism, precocious puberty, anal stenosis	Low serum IgM and IgG2 subclass, impaired response to vaccine antigens: poliomyelis, measles, hepatitis B viruses, and to pneumococci, lymphopenia, low switched memory B cells and CD8 cytotoxic T cells

## 2 Combined immunodeficiencies

### 2.1 Ataxia telangiectasia

Molecular pathology: Ataxia telangiectasia (AT), a.k.a. Louis–Bar syndrome or Boder–Sedgwick syndrome, is an autosomal recessive disorder characterized by a DNA repair defect and chromosomal instability, syndromic immunodeficiency, cerebellar ataxia, telangiectasia, radiosensitivity, and susceptibility to malignant transformation. AT is caused by biallelic germline pathogenic variants in the *ataxia-telangiectasia mutated* (*ATM*) gene, located at 11q22.3-23.1 and encoding the serine/threonine phosphatidylinositol 3-kinase protein. ATM is a predominantly nuclear enzyme engaged in cell cycle checkpoint and acting as a sensor and effector of the DNA DSB repair, as the major and the earliest kinase to be activated in the cellular response to DNA damage (13–15). The ATM signaling activation comprises two main pathways: canonical, in which DNA DSB, including V(D)J rearrangement and immunoglobulin class switch recombination, starts the recruitment of the MRE11–RAD50–NBS1 complex, stimulating ATM activity, and non-canonical, associated with cellular mitochondrial oxidative stress, insulin metabolic regulation, and synaptic transmission (15–17). Upon detection of DNA damage, the MRN complex is congregated and acts as an adaptor for ATM autophosphorylation on serine 1981 and dissociation of its inactive dimers into active ATM monomers, which is a fundamental process for signal spreading via H2AX histone phosphorylation (15–17). Whereas ATM is a master of H2AX phosphorylation in response to DNA DSB, members of the phosphatidylinositol 3-kinase (PI3K) family, such as DNA-dependent protein kinase (DNA-PK), ATM, and Rad3-related (ATR), are involved in this pathway.

Importantly, ATM signaling is critical for DSB repair mechanisms in V(D)J recombination and T cell and immunoglobulin B cell receptor formation and class switch recombination (CSR) in mature B cells, implicating its role in vital immune functions.

Immune deficiency and immune dysregulation: The multiple mechanisms giving rise to combined cellular and humoral immune deficiency in AT are primarily associated with the ATM dysfunction in DNA DSB repair (18, 19). Chromosomal translocation in the regions of chromosomes 7 and 14, which are associated with T cell receptors and heavy chain immunoglobulin coding regions, has also been postulated to be involved in the immunodeficiency in AT (18). Moreover, a dysregulated ATM response to oxidative stress in AT may contribute to defective thymic development due to apoptosis and reduced bone marrow hematopoiesis (20). Low T-cell receptor excision circle (TREC) and kappa-deleting excision circle (KREC) profiling in AT-affected individuals diagnosed on newborn screening points to the multifactorial and complex etiology of decreased thymic and bone marrow output (21–23). The impaired TCR and BCR recombination and CSR processes result in a deficiency in cellular and humoral immune responses. In a significant proportion reaching 70% of AT patients, lymphopenia occurs already at diagnosis and tends to progress over time. The most prominent feature of cellular immunodeficiency is a defective lymphocyte neogenesis with low numbers of T CD3+ cells, in particular, T CD4+ naïve helper cells, low CD4+CD45+RA+:CD4+CD45RO+ ratio, low CD4+CD45RO+CD127-CD25++ regulatory cells, as well as low B CD19+ cells (24, 25). Abnormal T and B cell differentiation and maturation were demonstrated by flow cytometric immunophenotyping, with depleted unswitched memory/marginal zone-like and switched memory B cells, and a decrease in T CD4+ and T CD8+ central and effector memory cells (19, 26). Disturbed development of switched memory B cells, impaired DNA repair pathway in immunoglobulin class switching, and defective germinal center reaction result in humoral immunodeficiency. It is observable in all AT patients in variable degree, but the defective CSR pathway has a discriminatory role and is reflected by the hyper-IgM phenotype characterized by a higher risk for more profound immunodeficiency, infections, immune dysregulation with autoimmune and granulomatous complications, higher risk of malignancy, and reduced survival (19, 26, 27). It has also been demonstrated that IgA deficiency is associated with lower lymphocyte counts and poorer prognosis; hence, it may be used as a simple surrogate predictive marker (28). Importantly, the deficiency of unswitched memory/marginal zone-like B cells and reduced levels of IgG2 (26, 28) subclass particularly make AT-affected patients susceptible to *Streptococcus pneumoniae* and *Haemophilus influenzae* recurrent and chronic respiratory infections (2, 26). Immune dysregulation, manifesting as autoimmune disorders, such as neutropenia (AIN), autoimmune hemolytic anemia (AIHA), juvenile idiopathic arthritis (JIA), organ-specific immunopathology presenting as chronic hepatitis and cutaneous and visceral pulmonary, hepatic granulomatosis, had a striking predilection to the hyper-IgM (HIGM) phenotype (26). Non-effective antibody maturation, expansion of IgM autoantibodies during immune response to infections, and an increased germinal center autoreactive B cell population were proposed as contributing factors to immune dysregulation in HIGM AT (29, 30).

Malignancy: The susceptibility to malignant transformation in AT patients is closely related to the molecular pathology of ATM, both nuclear with defective DNA DSB repair and cell cycle checkpoint regulation and cellular with oxidative stress and triggering apoptosis. Abnormal V(D)J pathway and T and B cell receptor gene rearrangements disturbing the generation of mature lymphocyte antigenic receptors, depletion of T and B cell compartments, and deficiency in the CSR process contribute to the hyper-IgM phenotype as well as IgA and IgG2 subclass deficiencies. Failure in cellular and humoral immune responses is associated with impaired surveillance and increased risk of oncogenesis (31, 32). Beyond the ATM functions in DNA DSB repair, it also plays a role as a modulator of the innate immune response and cytokine signaling, such as activation of Nuclear Factor kappa B (NFκB) canonical pathway and NFκB essential modulator (NEMO) phosphorylation, driving oncogenic miRNA expression (32, 33). In addition, AT patients present with invariant natural killer T cell (iNKT) deficiency that participate in controlling viral infections. It was, therefore, hypothesized that Epstein–Barr virus (EBV) latency may contribute to EBV-related lymphoproliferative disease and lymphomagenesis (34). The cumulative incidence of cancer in children has been estimated to be 25% (31) and 21.7% in pediatric AT patients by the age of 15 years, with non-Hodgkin lymphoma being the most common malignancy (35). Most frequently, large B cell lymphoma was reported in patients at every age (36–38), and less frequently, T cell lymphoblastic leukemia (T-ALL) or Hodgkin lymphoma (35), and solid tumors of the central nervous system, such as medulloblastoma, craniopharyngioma, glioma (39) in children, with an increased incidence of breast, ovarian, gastric, pancreatic, and colorectal carcinomas in adult patients (31). Expert consensus recommendations on cancer screening and surveillance highlight challenges of cancer management in AT patients due to preexisting organ immunopathology and dysfunction, excessive toxicity of the therapy, and radiosensitivity, which may directly translate into the outcomes of malignant disorders (40–43).

### 2.2 Nijmegen breakage syndrome

Molecular pathology: Nijmegen breakage syndrome (NBS) is a recessively inherited syndromic immunodeficiency and cancer predisposition disorder associated with chromosomal instability and a defect in DNA DSB repair. The molecular background of NBS is associated with the regulatory function of the complex of meiotic recombination 11 homolog1 (MRE11), ATP-binding cassette-ATPase (RAD50), and phosphopeptide-binding Nijmegen syndrome protein 1 (NBS1). The MRE11–RAD50–NBS1 (MRN) complex is one of the earliest responders to DNA DSB (44, 45). The principal structural, enzymatic, sensing, and signaling roles of MRN in the recognition and stabilization of DNA DSB, cell-cycle checkpoint signaling cascade, and regulation of chromatin remodeling are promoted by its 3-protein multidomain composition. RAD50 component promotes long-range allostery through its coiled-coil and zinc-hook domains. The dual exo and endonuclease biological functions of MRE11 in DSB are to organize the MRN complex architecture and bind RAD50, NBS1 with DNA, and DNA end processing (46). The principal role of the MRE11 component in DNA repair machinery is reflected by the pathophysiology of ataxia–telangiectasia-like disorder (ATLD) caused by pathogenic missense or nonsense variants in the *MRE11* gene (47). In hitherto reported patients with biallelic loss-of-function variants in the *RAD50* gene classified as NBS-like disorder, common phenotypic features with NBS were observable, e.g., facial dysmorphism, prominent forehead, microcephaly, and short stature, along with bone marrow failure and immunodeficiency (48, 49).

The role of the MRN in the DSB repair is multidirectional as the complex senses, processes, signals, and directs DSB strategy to homologous recombination (HR) or non-homologous end joining (NHEJ). The involvement in chromatin remodeling encompasses promoting stalled replication forks degradation, telomere maintenance, initial DNA resection, and preventing senescence in mitotically active cells (50). The complexity of MRN functions in DSB and cell-cycle checkpoints is therefore crucial in the genomic integrity and cellular homeostasis, thereby underscoring the role of its alterations in immune, lymphoproliferative, and neurological disorders in NBS.

Immune deficiency and immune dysregulation: The MRN complex is a keystone factor initiating and activating the DNA DSB pathway, which occurs physiologically during V(D)J and class switch recombination. Variants in the *NBN* gene causing defective V(D)J recombination process result in a reduction in the B cell bone marrow output, reduced BCR repertoire, and antibody deficiency. Importantly, the loss of juxtaposed Ig genes can lead to a higher risk of aberrant rearrangements and thereby high susceptibility to lymphomagenesis in NBS (51). Low TREC and KREC numbers are detected by neonatal screening, thereby being potentially useful in early life diagnosis of the disease (52, 53). Flow cytometric peripheral blood lymphocyte immunophenotyping in pediatric NBS patients demonstrated a reduced number of B cells and their maturational defect (54). T-cell dependent B cell differentiation pathway was the most severely impaired, resulting in the defective production of memory B cells. A poor T cell help contributed also to the aberrant class switch recombination process, causing an increased proportion of the IgM-only memory B cells and an inadequate production of IgG and IgA immunoglobulin isotypes (54). Within the T cell compartment, a reduced number of total T lymphocytes and their aberrant maturation were demonstrated, with low numbers of CD4+CD45RA+CD31+ recent thymic emigrants (RTE) and both naïve CD4+ T helper and CD8+ T cytotoxic/suppressor cells, indicating that variants in *NBN* severely affect the thymic production of T cells (53). Functional impairment of T cells with their increased proportion at the terminal differentiation state was also demonstrated. Immunosenescence reflected by CD57 and an immune checkpoint receptor, killer cell lectin-like receptor G1 (KLRG1), and an exhaustion marker, programmed death 1 receptor (PD-1), was expressed by CD4+ and CD8+ cells of NBS patients, but they did not show the expression of a co-stimulatory CD28 molecule (55). The aberrant T and B lymphocyte developmental pathways might explain the increased susceptibility of affected patients to both recurrent, severe opportunistic infections (2) and malignant transformation (56).

Malignancy: In patients with NBS, the cumulative cancer incidence by the age of 10 years was estimated to be 42% and dramatically increases further, even up to 78% by the age of 20 years. The mortality rate in those who develop malignancies ranges from 22 to 67%, with poor prognosis largely depending on severe infectious and autoimmune complications and the low serum IgG levels predisposing to infectious and inflammatory disorders (57). The exceptionally high susceptibility to malignant transformation in NBS can be attributed to several factors, such as the germline p.I171V variant which may be considered a risk factor for childhood acute lymphoblastic leukemia (ALL) (58), impaired telomeric repair which synergizes with telomere attriction and dysfunction (59), as well as an Epstein–Barr virus (EBV) infection and increased IgM serum levels as these features characterized patients who developed B cell non-Hodgkin lymphoma (B-NHL) (58). An impaired mitochondrial homeostasis and oxidative stress with a lower total antioxidant capacity and overproduction of reactive oxygen species, resulting in oxidative damage of proteins, lipids, and DNA, has been hypothesized to be a contributing factor to carcinogenesis and a biomarker of the disease (60).

Due to the aberrant V(D)J recombination in NBS, impaired resolution of RAG-induced immunoglobulin heavy chain locus (IgH) breaks may promote complex translocations involving this region in lymphomas (61). Non-Hodgkin lymphoma is the most commonly diagnosed malignancy in NBS (61–63). There is a strong predominance of diffuse large B cell lymphoma (DLBCL) and T cell lymphoblastic lymphoma (TLBL/ALL), and their distribution in NBS patients is almost equal, achieving 55% and 45%, respectively (61). Interestingly, peripheral T cell lymphomas (PTCL) diagnosed in patients with NBS are rarely observable in the non-syndromic pediatric population (64). Other subtypes of lymphomas include Hodgkin lymphoma, with frequent gastric extranodal site (65), or Burkitt-like lymphoma. The diagnosis of malignancy is often delayed due to nonspecific symptomatology, such as fever or lymphadenopathy, which appear to be infection-related, and consequently, an advanced stage and multiorgan involvement of lymphoma is common. NBS-affected patients, in particular, those who were previously treated because of B cell lymphoma, show a high risk, reaching 33% of them developing secondary malignancy, such as lymphoma and acute lymphoblastic or myeloblastic leukemia (ALL or AML) (61). While lymphoid malignancies predominate in NBS, solid tumors such as neuroblastoma, medulloblastoma, glioma, rhabdomyosarcoma, ovarian cancer, and thyroid and gastric carcinoma were less frequently reported (66–68).

### 2.3 Bloom syndrome

Molecular pathology: Bloom syndrome (BS) is an autosomal recessive disorder characterized by a spectrum of phenotypic features, including growth retardation, facial dysmorphism, cutaneous radiosensitivity, insulin resistance, immunodeficiency, and predisposition to malignancy. It is caused by a loss-of-function homozygous or compound heterozygous variants in the (*BLM*) gene located at 15q26.1 and encoding the RecQL3 DNA helicase (69). The enzyme belongs to a highly conserved RecQL helicase family of DNA-dependent ATPases and ATP-dependent DNA unwinding enzymes (70). Human RecQ helicases are involved in various pathways of DNA metabolism, in the most important repair process, which is homologous recombination, in DNA replication, and in the stabilization and repair of damaged replication forks. *BLM* utilizes helicase activity for sister chromatid segregation in mitosis, in meiotic recombination, and also in telomere maintenance, preventing them from degradation (70, 71). *BLM* plays an essential role in the preservation of genomic stability and centromeric, telomeric, and ribosomal DNA sequences stabilization, as well as innate immunity homeostasis, acting at the interface between DNA replication, recombination, and repair (72). Therefore, BS is characterized by an increase in chromosomal aberrations, including chromatid gaps and breaks, telomere associations, and quadriradial chromosomes resulting from unsolved recombination between homologous chromosomes (72). Beyond functional abnormalities in BS, which can be explained by defective DNA damage response and genomic instability, it has also been hypothesized that oxidative stress, a common denominator for chromosomal instability syndromes, contributes to cellular pathology. *BLM*-deficient cells show high levels of reactive oxygen species and oxidative DNA damage and reduction of DNA replication speed (73). Impaired mitochondrial homeostasis, including abnormalities in structure and function, and mitochondrial damage result in chronic oxidative stress, contributing to genome instability, loss of cell viability, and nuclear DNA mutations initiating the tumor cell phenotype (73, 74).

Immune deficiency and immune dysregulation: The role of *BLM* and cellular functions of its product, ReQL3 helicase, in DNA replication, repair, and transcription is a link between chromosomal instability, immune cell functions, and phenotypic features such as recurrent otitis media and pneumonia, immunodeficiency, and susceptibility to carcinogenesis in BS (75). Similarly to other syndromes associated with defective DNA repair, it determines the abnormal development of T and B cell antigenic receptors and the V(D)J recombination as well as somatic hypermutation and class switch recombination processes involved in immunoglobulin production. In BS patients, flow cytometric analysis of the B and T cell compartments demonstrated decreased absolute numbers of T cells, reduced CD4+ T helper cell subset, and low CD4+ and cytotoxic/suppressor CD8+ naïve, effector memory, and central memory T cells (75, 76). Maturational defect within the B cell compartment was also shown with the reduced numbers of transitional and naïve mature, memory, and natural effector B cells that correlated with low IgG, IgM, and IgA serum levels (75). Whereas the median frequency of SHM in transcripts for the heavy constant gamma chain of IgG (IGHG) and IgA (IGHA) in naïve B cells was within the normal range, class switching to downstream constant regions and replacement mutations in the complementarity determining regions (CDRs) of antibodies, increasing the affinity for the antigen, was reduced in BS patients (75, 77). It may therefore be assumed that dysfunctional DNA ReQL3 helicase activity in BS, resulting in genomic instability, disturbs T cell development and impairs T cell help to promote CSR and antigen affinity maturation in B cells. Reduction in the DNA replication rate, impaired cell cycle progression, and oxidative DNA damage contribute to the dysregulation of cellular processes, which negatively affect the proliferation pathway, resulting in a decreased proliferation rate, thereby causing immunodeficiency in BS (78).

Malignancy: The chromosomal instability and subsequent somatic mutations resulting from this instability are the major factors determining the high risk of malignancy in BS individuals. It is worth noting that the median age of cancer diagnosis in pediatric patients with BS is 12 years (79). The most frequently recognized malignancies are lymphatic neoplasms, lymphoblastic and myeloid leukemia, and lymphoma (80), as well as the second most common cancer in BS patients, colorectal cancer, with an early onset at 16 years (81). There is a multiplicity of cancer types encountered in pediatric BS patients, and about 35% of the affected patients have multiple cancers, including gastrointestinal, genital, and urinary tract carcinoma, Wilms tumor, medulloblastoma, osteosarcoma, and retinoblastoma (82). Solid tumors, such as breast cancer, non-small cell lung cancer, colorectal and oropharyngeal cancers, or hematologic malignancies were diagnosed in young adult BS patients (83, 84). Cancer surveillance and screening programs are necessary for BS patients due to the high risk and young age of neoplastic transformation. Awareness of cancer-related symptoms, such as abnormal growths or nodules, lymphadenopathy, rectal bleeding, chronic pain, fatigue, weight loss, and fever, which are the warning signs of potential malignancy, is required for caring physicians and BS patients’ families (82, 84). Due to the aberrant DNA repair, oxidative stress, and subsequent significant risk of chemotherapy-related toxicity, dose reduction regimens are recommended for the therapy of cancer (40, 41, 79).

### 2.4 Postmeiotic segregation increased 2 deficiency

Molecular pathology: Postmeiotic segregation increased 2 deficiency (PMS2) belongs to a group of hereditary cancer predisposition syndromes caused by pathogenic biallelic homozygous or compound heterozygous germline variants in one of the post-replicative DNA mismatched repair (MMR) genes, *MUTL homolog 1* (*MLH1*), *MUTS homolog 2* and *6* (*MSH2, MSH6*), or *PMS2*. Referring to the underlying mechanism affecting the MMR system, the condition, constitutional mismatch repair deficiency (CMMRD) syndrome, is characterized primarily by the development of childhood-onset cancers (83). MMR is an essential process playing a role in maintaining genomic integrity by correcting single-base-pair mismatches and small misalignments, such as insertion–deletion loops, which arise during replication (85). The MMR machinery is not only involved in DNA repair and excision of base–base and large indel mismatches by a MLH1/PMS2 heterodimer, but its biological function is also an apoptotic response to DNA-damaging agents (86).

Immune deficiency and immune dysregulation: The MMR system functions to stabilize the genome by correcting errors generated during replication and to ensure that the fidelity of recombination is associated with its crucial antimutator role. Pathogenic variants in the *PMS2* gene predispose to DNA instabilities and are primarily linked to carcinogenesis. The MMR pathway involvement in class switch recombination and somatic hypermutation processes sheds light on its contradictory role as a mutator factor (87). It has been demonstrated that PMS2 deficiency is associated with impaired CSR pathway, thereby leading to an intrinsic switched and unswitched memory cell B and plasmablast aberrant generation (88, 89). Consequently, defective immunoglobulin maturational processes in PMS2-deficient patients were shown, with decreased serum levels of immunoglobulin IgG, IgG2, and IgG4 subclasses, IgA, and IgE isotypes, while the IgM levels were normal or mildly to moderately elevated. While in two yet asymptomatic patients with PMS2 deficiency antinuclear antibodies (ANA) were detected, observation toward immune dysregulation and development of a connective tissue disease or neuromuscular disorder is required. However, facing a small number of patients with PMS2 deficiency and a relatively high prevalence of ANA positivity (>10%) among pediatric and adolescent populations, concluding about predisposition to autoimmunity requires further studies (89, 90).

Malignancy: Pathogenic variants in genes relevant for the MMR pathway result in malfunction of the machinery to identify and excise DNA damage, while the MLH1–PMS2 complex is specifically involved in mismatch excision (91). Abrogation of the essential MMR genes is followed by abnormal base incorporation and indels, especially in microsatellite regions, resulting in an increased mutational burden and microsatellite instability, a hallmark of CMMRD tumors (92). The *PMS2* gene is the most affected among all MMR genes with cancer-predisposing variants, and PMS2-deficient patients develop devastating malignancies at an early age, with a median onset of about 7 years (92), and the earliest manifestations of childhood-onset cancers were T cell leukemia and giant cell glioblastoma at the age of 2 years and T cell non-Hodgkin lymphoma (NHL) at the age of 3 years (93).

### 2.5 Polymerase ϵ subunit 1 deficiency

Molecular pathology: DNA polymerase epsilon (Pol ϵ) contains an intrinsic proofreading exonuclease activity and is the major leading strand replicase during the replication of undamaged nuclear DNA in normal cells. It has also been proposed that Pol ϵ may proofread errors made by other polymerases, including mismatches made during DNA synthesis under stress (94, 95). The *POLE* gene encodes the catalytic and proofreading subunits of Pol ϵ, thereby playing an important role in DNA replication fidelity and genomic stability. *POLE* is a pleiotropic gene with heterogeneous phenotypic expression of pathogenic variants, depending on the type of variant, impact on the protein structure and function, and mode of inheritance (96). Heterozygous *POLE* pathogenic missense variants, which predispose to a cancerogenetic phenotype of polymerase proofreading-associated polyposis (PPAP), are located in the exonuclease domain of Pol ϵ and affect its proofreading function, increasing mispaired bases (97). Biallelic variants in *POLE1*, in turn, causing partial loss-of-function and markedly reduced protein expression, are associated with two syndromic phenotypes, with intrauterine growth restriction, metaphyseal dysplasia, congenital adrenal hypoplasia, and genitourinary abnormalities (IMAGe), and facial dysmorphism, immunodeficiency, livedo, and short stature (FILS), both related to impaired immune response and posing an increased risk of malignancy (97).

Immune deficiency and immune dysregulation: While most patients with IMAGe syndrome carry pathogenic gain-of-function variants in the *cyclin-dependent kinase inhibitor 1C* (*CDKN1C*) gene, a negative regulator of cell proliferation, *POLE1* has also been demonstrated to entail IMAGe syndrome characterized by intrauterine growth restriction, metaphyseal dysplasia, adrenal hypoplasia and insufficiency, and genitourinary abnormalities with distinctive facial gestalt. In all 15 affected individuals, one allele with the same intronic variant (c.1686 + 32C>G), resulting in the abnormal splicing of exon 15, was identified (98). The patients presented with respiratory tract infections, and one of them developed CMV pneumonia and EBV-triggered hemophagocytic lymphohistiocytosis. Immunodeficiency with variable T and B cell lymphopenia, NK cell deficiency, and hypogammaglobulinemia was also identified in this POLE1-IMAGe cohort (96–98). In 11 individuals belonging to one family affected with FILS syndrome, the (g.G4444 + 3 A>G) variant of intron 34 in the *POLE1* gene, resulting in abnormal splicing and impaired gene expression (99), was demonstrated, and further reports brought new insight into the FILS syndrome, adding (c.5811 + 2T>C) (100) and (c.100C>T) (101) to the spectrum of its genetic underpinnings. The immunodeficiency included decreased IgM and IgG2 subclass serum levels, lack of specific anti-polysaccharide antibodies, impaired B cell development with unswitched and switched memory B cell generation as well as low naive T cell counts and decreased T cell proliferation (99). Neither IMAGe nor FILS syndrome patients due to POLE1 deficiency were reported to present signs of immune dysregulation with autoimmunity or allergy.

Malignancy: Heterozygous pathogenic missense germline and somatic variants in *POLE* are associated with PAPP, particularly (p.Asn363Lys), (p.Val411Leu), and (p.Leu424Val), an autosomal dominantly inherited condition predisposing to colonic polyps, colorectal and endometrial cancers, and hypermutated, highly immunogenic tumors (102–104). However, LoF variants affecting DNA replication machinery and *POLE*-encoded polymerase ϵ1 catalytic subunit causing IMAGe and FILS syndromes do not seem to be associated with a higher risk for cancers.

### 2.6 Ring finger nuclear factor 168 deficiency

Molecular pathology: *Ring finger nuclear factor 168* (*RNF168*) gene encodes the ubiquitin E3 ligase RNF168 (a.k.a. RIDDLIN), whose enzymatic activity is crucial in collecting a set of reparatory proteins at the site of DNA damage. The DNA repair mechanisms rely on multiple regulatory and signaling processes, including histone ubiquitination, participating in chromatin modifications, amplifying the damage signals, and recruiting the DNA repair proteins (105). DNA DSB repair is initiated by the MRN complex binding to the broken DNA ends, which triggers recruitment and activation of ATM kinase, phosphorylating H2AX histone to form γH2AX. The RNF168 E3 ligase ubiquitinates histone H2AX at the N-terminus, and this process is essential to alter the chromatin conformation and form a docking site for mediator of DNA damage and checkpoint 1 (MDC1) to initiate the ubiquitin-dependent DNA damage response (105, 106). Variants in the *RNF168* gene are the genetic background for RIDDLE syndrome (radiosensitivity, immune deficiency, dysmorphism, learning difficulties), characterized by deficiency of RNF168 ligase and repair proteins, tumor suppressor p53 binding protein 1 (53BP1), and BRCA1 recruitment to sites of DSB, resulting in hypersensitivity to ionizing radiation and cell cycle checkpoint defects (107–109). This phenomenon shed light on the ubiquitination (Ub)-DDR pathway that is controlled by MDC1 and RNF168 (109).

Immune deficiency and immune dysregulation: The flawed DNA DSB pathways are associated with an immunophenotype showing impaired V(D)J recombination and class switch recombination processes in lymphocytes, as well as cellular radiosensitivity and increased risk of carcinogenesis. These chromosome instability syndromes often share overlapping immune and non-immune features, being partial phenocopies of one another and thereby termed as XCIND disorders (X-ray sensitivity, cancer susceptibility, immunodeficiency, neurological abnormalities, and DNA DSB repair dysfunction) (108). There was no evidence of an affected V(D)J pathway and abnormalities within the B and T cell compartments in patients with RIDDLE syndrome. Immunoglobulin G and A deficiency, accompanied by an increased IgM level reflecting defective CSR process and isotype switching due to a failure of the DNA DSB pathway, was demonstrated (110, 111). Phenotypic features of immune dysregulation, such as allergy or autoimmunity, were not recognized in patients with RNF168 deficiency.

Malignancy: Whereas an increased risk of cancer is a hallmark of DNA DSP repair defects and is presumed to be a cardinal feature of the defective Ub-DDR pathway, in a very small group of hitherto reported patients with RNF168 deficiency, malignant transformation was not observable (109–111).

### 2.7 DNA ligase I deficiency

Molecular pathology: Three genes, *LIG1*, *LIG3*, and *LIG4*, encode ATP-dependent DNA ligases, LigI, LigIII, and LigIV, respectively. Ligase I is a nuclear enzyme and shows a significant functional redundancy with LigIII in DNA replication, base excision and nucleotide excision repair, and single-strand break repair. Steady-state levels of LigI correlate with cell proliferation, and its activity is associated with the replication machinery; hence, LigI excision repair pathway occurring during the S phase of the cell cycle is likely closely linked with DNA replication. DNA mismatch repair or recombinational repair of DNA double-strand break pathways associated with DNA replication makes LigI a predominant ligase in maintaining the genome integrity (112, 113). This enzyme also has unique functions in post-replicative repair, including the generation of sister chromatid telomere fusions (114). Additionally, it is a key component of DNA methylation machinery as its N-terminal region is involved in phosphorylation-regulated protein–protein interactions (114). While LigI plays an essential role in nuclear DNA replication and DNA repair pathways, other functionally overlapping DNA ligases can compensate for the loss of LigI in cells showing one functional allele. Noticeably, biallelic hypomorphic variants in the *LIG1* gene affecting DNA ligation and Mg^2+^-dependent DNA binding result in a spectrum of immunodeficiency syndromes (115).

Immune deficiency and immune dysregulation: LigI immune deficiency syndromes are a group of rare disorders caused by biallelic pathogenic variants in the *LIG1* gene (116). It was demonstrated that the spectrum of clinical and immunological phenotypes, as well as variable severity of immune deficiency in affected patients, were related to different genotypes. The phenotypic presentation ranged from normal growth to early growth failure, yet dysmorphology and neurodevelopmental disorders were not observed (116–118). The immunodeficiency was characterized most frequently by hypogammaglobulinemia, which in two of six hitherto reported patients led to the initial diagnosis of common variable immunodeficiency (CVID), but two rare variants in the *LIG1* gene were subsequently identified in them (c.1922G>t, p.R641L and c.1244delC, p.T415Mfs*10) (116). Four children presented profound lymphopenia with T-B-NK+ immunophenotype, defective response to mitogens, and increased proportion of γδ T cells. They were diagnosed with severe combined immunodeficiency (SCID) (116–118) and underwent HSCT. Among them, one infant bearing two novel compound heterozygous variants in *LIG1* (c.2312 G>A, p.Arg771Gly and c.776 + 5G>T, p.Pro260*) had Omenn-like syndrome with exfoliative erythrodema and lymphadenopathy (118).

Malignancy: While the consequence of LigI deficiency is defective DNA repair and replication, an increased risk of malignancy in affected patients should be considered, although a small number of hitherto reported patients pose difficulties in the precise determination of long-term disease sequelae.

### 2.8 NSMCE3 deficiency

Molecular pathology: The *non-structural maintenance of chromosome element 3* (*NSMCE3*, a.k.a. *NDNL2* or *MAGEG1*) gene encoding SMC5/6 subunit, a member of the melanoma-associated antigen (MAGE) protein family, participates in large-scale changes in genome reorganization through different processes other than canonical chromatin/histone-modifying enzymes (119). The structural maintenance of chromosomes (SMC) is enzymatic protein complexes that play a unique role in the interphase regulation of chromatin folding. The process occurs independently of nucleosomes or other enzymes involved in chromatin compaction, and SMC complexes are an important part of genome homeostasis (119). In particular, the SMC5/6 subunit plays important cellular functions of growth and genotoxin resistance and is essential for DNA damage response and chromosome segregation (120). Homozygous or compound heterozygous missense variants in *NSMCE3* cause the disruption of interactions within the SMC5/6 complex and lead to its instability. In cells of the affected patients, chromosomal rearrangements, micronuclei, sensitivity to replication stress and DNA damage, and defective homologous replication were demonstrated, thereby expanding the spectrum of chromosome breakage syndromes (121).

Immune deficiency and immune dysregulation: The phenotype of missense variants in *NSMCE3* is associated with a rapidly progressive illness, lung disease immunodeficiency chromosome breakage syndrome (LICS), linked to severe pneumonia and respiratory failure in early childhood (121–124). The immunological features in patients bearing variants in *NSMCE3* were diverse, with normal immunoglobulin G, A, and E isotypes but increased IgM levels, normal B cell numbers, and yet impaired specific antibody response to pneumococcal vaccine. Within the T cell compartment, low T helper CD4+ and T cytotoxic-suppressor CD8+ were demonstrated, along with defective T cell proliferation to mitogens, phytohemagglutinin, concanavalin A, pokeweed, and, in particular, to tetanus toxoid and *Candida* (121).

Malignancy: In LICS due to variants in *NSMCE3*, malignant transformation was not diagnosed in any of the affected individuals. However, it needs to be highlighted that the fatal course of the disease in early childhood due to respiratory failure in hitherto reported patients rendered a long-term observation not possible (123, 124). Nonetheless, like other chromosome instability syndromes, the disease can pose an increased risk of malignant transformation due to increased levels of chromosomal instability, impaired recovery from replication stress, cellular radioresistance, proliferation, and survival (124).

### 2.9 Immunodeficiency, centromeric instability, and facial anomalies syndrome

Molecular pathology: Immunodeficiency, centromeric instability, and facial anomalies (ICF) syndrome is a rare, autosomal recessively inherited disorder characterized by multiradial chromosomal configurations involving the pericentromeric regions of chromosomes 1, 9, and 16 (125) and a heterogeneity of causative gene variants. The first causative gene identified for the ICF type 1 syndrome was *DNA methyltransferase 3 beta* (*DNMT3B*), encoding an enzyme crucial for establishing new DNA methylation patterns during early development, working in epigenetic regulation of gene expression, and maintaining chromosomal stability (125). Other genes involved in the methylation process of pericentromeric satellite repeats and linked to the pathogenesis of the ICF syndrome are *zinc finger and BTB domain containing 24* (*ZBTB24*), *cell division cycle associated 7* (*CDCA7*), and *helicase lymphoid specific* (*HELLS*), associated with ICF types 2, 3, and 4, respectively (126). DNA methylation predominantly occurs at the C5 position of cytosine bases in the CpG context and plays a crucial regulatory role in gene transcription and repression. Whereas pathogenic variants in *DNMT3B* result in hypomethylation at distinct pericentromeric and subtelomeric regions, variants in ZBTB24, CDCA7, and HELLS, disrupting replication–uncoupled maintenance DNA methylation, occur in pericentromeric and centromeric regions (127). In the ICF syndrome, DNA instability accompanied by hypomethylation manifests in lymphocytes as stretched heterochromatin, chromosome breaks, and multiradial configuration involving the pericentromeric regions of chromosomes 1, 9, and 16 (125).

Immune deficiency and immune dysregulation: The abnormal gene methylome, aberrant transcript initiation, and hypomethylation of heterochromatin explain the developmental disorders, clinical features of immunodeficiency predominantly associated with recurrent respiratory tract infections, and the immunological phenotype in ICF. Hypo- or agammaglobulinemia with marked reduction of all immunoglobulin isotypes and IgG subclasses demonstrated in ICF patients may result from impaired lymphogenesis, deficient CSR process in B cells, defective switched memory B cell generation, and abnormal naïve B cell activation (126–129). Abnormalities in the T cell compartment included reduced total CD3+ T cell, decreased CD4+CD45RA+CD31+ recent thymic emigrant, naïve T, CD4+ T helper cell, CD8+ cytotoxic-suppressor T cell, and CD56+ CD16+ NK cell counts, as well as decreased CD4+:CD8+ T cell ratio. A skewed Th2-immune response with a reduced T regulatory cell count and an expanded follicular T cell subset were also observable (130). The immunophenotype with deregulated immunoglobulin signaling in B cells and imbalance in T cell subsets correlates with immune dysregulation, such as autoimmune cytopenia, hepatitis, nephritis, and thyroiditis in ICF patients (131).

Malignancy: The helicase chromatin remodeler HELLS, which is involved in the pathogenesis of ICF4, plays a crucial role in DNA repair, genome stability, and multiple cancer-associated pathways associated with cell death and survival, sustaining proliferative signaling, and regulation of oncogenic and tumor suppressor factors (132, 133). HELLS is implicated in many types of malignancies, such as retinoblastoma, glioblastoma, colorectal cancer, and hepatocellular carcinoma (133). Reports also point to the involvement of HELLS in leukemogenesis and lymphomagenesis due to the high incidence of alternative mRNA transcripts (134). Allogeneic hematopoietic stem cell transplantation is the only curative treatment, and among pediatric ICF patients with all four genotypes, myelodysplasia/hematological malignancy or immune dysregulation accounted for 17% and 22% of HSCT indications, respectively (135).

## 3 Severe combined immunodeficiency

### 3.1 DNA ligase IV deficiency

Molecular pathology: DNA ligase IV deficiency or LigIV syndrome is inherited as an autosomal recessive inborn error of immunity characterized by the heterogeneity of clinical features and individual patient immunophenotype ranging from mild deficit in immunity to T-B-NK+ severe combined immunodeficiency and lymphoid malignancy (10). Beyond immunodeficiency, the affected patients show microcephaly, growth failure, skin abnormalities, and developmental delay (136–138). The syndrome is caused by hypomorphic pathogenic variants in the *LIG4* gene encoding an enzymatic protein playing a crucial role in V(D)J recombination and in the non-homologous end-joining (NHEJ) DNA repair pathway that is an essential mechanism of DSB repair (139, 140). LigIV establishes complexes with XRCC4 at the site of DNA damage, where it acts in the final step of NHEJ, ligating the DNA strands together (138, 140). Noticeably, all variants in *LIG4* reported in patients with LigIV syndrome are hypomorphic. They are either positioned close to the enzyme’s active catalytic site within the conserved ligase motif, preserving 5%–10% residual adenylation activity and DNA double-strand joining activity, or within the core of the N-terminal domain, altering the beta-sheet structure and the ATP-binding pocket (141).

Immune deficiency and immune dysregulation: The NHEJ repair pathway engages LigIV in the V(D)J recombinational process in the B and T cell receptor generation. However, marked heterogeneity of the clinical phenotype and varying degrees of immunodeficiency with B and T cell lymphopenia have been reported in Lig4 syndrome, and most affected patients do not fulfill the criteria of SCID according to the IUIS classification (10). In pediatric patients with severely compromised immune response, recurrent pneumonia, gastroenteritis, urinary tract infection, sepsis, and encephalitis caused by pathogenic bacteria such as *Pseudomonas aeruginosa* and *Acinetobacter baumanii*, as well as *Pneumocystis jiroveci* and viruses, were reported (142, 143), with bronchiectasis as an early complication of respiratory tract infections. Hypo- and agammaglobulinemia and profound B cell lymphopenia, in particular low switched memory B cells, as well as severely impaired generation of T cells, both CD4+ and CD8+ subsets, impaired the proliferative response to mitogens, and NK cell deficiency was demonstrated in LigIV deficiency syndrome (136, 138, 142–144). Progressive bone marrow failure, with thrombocytopenia and neutropenia, and gradual pancytopenia were also observable in a proportion of patients. Interestingly, novel monoallelic missense variants in the LIG4 gene, causing haploinsufficiency, were characterized by immune dysregulation, clinically manifesting as pneumonitis, autoimmune cytopenia, granulomatous parotitis, and lymphoproliferation manifesting as lymphadenopathy and splenomegaly. The immunophenotype showed reduced counts of T CD4+CD127^low^CD25^hi^ regulatory cells that displayed a proinflammatory and activated phenotype (145).

Malignancy: The severity of the clinical, hematological, and immunological phenotype in LigIV deficiency syndrome seems to be a consequence of the type of variant and correlate with the subsequent protein truncation and residual enzymatic function of ligase. Beyond variable immunodeficiency, including T-B-NK+ SCID, all patients with LigIV deficiency syndrome presented with radiosensitivity, an additional risk factor to develop malignant transformation. Hematological malignancies, such as diffuse large B cell lymphoma localized in the lung, brain, nasopharynx, and palate and also acute lymphoblastic or acute myeloblastic leukemia, were reported in affected individuals (142, 146, 147). Reduced intensity conditioning regimen followed by hematopoietic stem cell transplantation offers a curative immune reconstitution.

### 3.2 Cernunnos/XLF (XRCC4-like factor) deficiency

Molecular pathology: Cernunnos or X-ray repair cross-complementing 4-like factor (XRCC4-like factor, XLF) is encoded by the *NHEJ1* gene and plays a key role in the non-homologous end-joining repair pathway of DNA DSB, promoting the ligation of broken noncomplementary DNA ends (148). NHEJ does not require a homologous template, may function in phases of the cell cycle, and yet is a highly error-prone process. It engages a variety of interacting protein factors, the most essential of which are Ku70/86 heterodimer, DNA–protein kinase catalytic subunit (DNA-PKcs), Artemis, XRCC4, XLF, and ligase IV (148). The NHEJ protein complex is also involved in the antigen receptor gene rearrangement in the joining steps of V, (D), and J segments, thereby contributing to antibody diversity. Variants in NHEJ1 resulting in mutant XLF alleles show reduced nontemplated nucleotide addition during V(D)J recombination, ultimately leading to defective B and T cell development (148). In patients affected with Cernunnos deficiency, disease-causing variants are located in the globular head of the XLF protein, interacting with XRCC4 and the coiled-coil stalk domain. The most common are nonsense variants, followed by missense and splicing, frameshift variants, and large deletions, yet there is no clear correlation between the type and location of the variant and the clinical presentations or severity of the phenotype (149). The clinical features include microcephaly, growth retardation, and facial dysmorphism, as well as radiosensitivity and immunodeficiency (149–152).

Immune deficiency and immune dysregulation: Variants in *NHEJ1* and Cernunnos/XLF deficiency impair the rejoining of the programmed DNA DSB during V(D)J recombination and result in the failure of T and B cell maturation and T-B-, but NK+ SCID immunophenotype, as these cells do not undergo the V(D)J recombination (150–153). Interestingly, some cases do not exhibit a B-cell deficiency (149, 154). Lymphopenia is the most frequently reported hematological disorder among affected children, but its severity may vary among cases, as alternative DNA repair proteins may operate in DNA DSB and lymphocyte antigen receptor gene rearrangements (150). The variability of immunoglobulin deficiency reflects B cell deficits in number and function, including a defective CSR process. The T cell compartment was characterized by T CD+ and CD8+ lymphopenia, low T cell receptor excision circle (TREC), and impaired response to mitogens (149–154). A restricted TCR repertoire and abnormal peripheral tolerance may contribute to autoimmunity cytopenia, most frequently autoimmune cytopenia, such as hemolytic anemia (150, 154). Due to the complexity of immunogenetic underpinnings of XLF deficiency, ranging from SCID through CID to immunocompetent individuals (155), different therapeutic options are used according to the immunological and clinical phenotype. These include immunoglobulin replacement, antimicrobial prophylaxis with antibiotic or cotrimoxazole and filgrastim in the case of neutropenia, but the only curative therapy for XLF deficiency-related SCID is HSCT following a reduced intensity conditioning chemotherapy regimen (150, 151, 153, 156).

Malignancy: While Cernunnos/XLF is a key player in the DNA DSB repair pathway, variants in *NHEJ1* result in disrupted chrosomal stability and render the patients affected with Cernunnos deficiency prone to malignant transformation. Diffuse large B cell lymphoma of the brain has been hitherto reported in one child with Cernunnos deficiency (157).

### 3.3 Protein kinase catalytic subunit deficiency

Molecular pathology: The DNA-dependent protein kinase is a serine/threonine protein kinase composed of a large catalytic subunit (DNA-PKcs) and the Ku70/86 heterodimer. It is a crucial component of the NHEJ repair of DNA DSB that maintains genomic integrity and is an essential factor of immune processes, V(D)J recombination, and CSR in lymphocyte maturation (158–160). DNA-PKcs acts as a sensor of DSB and, along with Ku70, Ku86, LigIV, XRCC4, Cernunnos/XLF, and Artemis, forms a seven-protein complex required for NHEJ (148, 158). It has also been demonstrated that DNA-PKcs shares a structural and functional similarity with ATM, which is an important element of the DNA DSB machinery and plays a more important role in the cell cycle checkpoint. Importantly, DNA-PKcs and ATM undergo complex regulations through autophosphorylation and mutual phosphorylation and show an overlapping activity in protein phosphorylation (161). Thus far, seven patients bearing disease-causing variants in the *PRKCD* gene have been reported, and among them, six patients shared Turkish ethnicity and carried the founder hypomorphic missense L3062R variant (162). All of the DNA-PKcs-deficient children presented with a complex immunological phenotype, whereas non-immunological features, predominantly limited to the nervous system, such as neurodevelopmental delay, seizures, and microcephaly, were less frequent (162).

Immune deficiency and immune dysregulation: Severe respiratory and digestive tract infections, most frequently bacterial but also opportunistic with *Candida*, *Aspergillus*, and non-tuberculous *Mycobacteria*, correlated with immune deficiency in the affected patients. Whereas the DNA-PKs deficiency is an extremely rare but severe disease with a predominantly hyperinflammatory phenotype, typical T-B-NK+ SCID was rarely present (162, 163). Immune dysregulation disorders included cutaneous, hepatic, and splenic granulomatosis, arthritis, and autoimmunity with the presence of anti-DNA, anti-TPO, anti-TG, anti-GAD65, anti-CaSR, and anti-cytokine (IL-22, IL-17A, IL-17F, IFN-α, IFN-λ, IFN-ω) autoantibodies due to impaired autoimmune regulator (AIRE) expression (163). Immunodeficiency was characterized by low thymic output, T CD4+ and T CD8+ lymphopenia, reduced naïve CD45RA+CD4+ T cells, impaired response to mitogens, restricted TCR repertoire, low B cells, IgA deficiency, and variable serum levels of IgG and IgM isotypes (162, 163). HSCT with reduced toxicity is the only curative therapeutic procedure for DNA-PKcs deficiency, offering T cell reconstitution, albeit preexisting autoimmune and inflammatory manifestations are a negative prognostic factor, posing the need to increase the experience of HSCT for patients with atypical disease (162).

Malignancy: EBV-induced non-Hodgkin lymphoma was hitherto noticed in one patient with DNA-PKcs deficiency. Further studies on potential new clinical cases might help to estimate the risk of carcinogenesis related to *PRKCD* variants.

### 3.4 Artemis deficiency

Molecular pathology: Artemis is encoded by the *DNA Cross-Link Repair 1C* (*DCLRE1C*) gene and is a key component of the seven-protein DNA DSB repair NHEJ complex composed of Ku heterodimer, Cernunnos/XLF, XRCC4, DNA-PKcs, LigIV, and Artemis. NHEJ is the most widely occurring DNA DSB repair during most phases of the cell cycle, and Artemis endonuclease activity is essential for trimming of broken DNA ends to make them suitable for joining by ligation (164). Artemis enzymatic protein belongs to the metallo-β-lactamase superfamily and shows associated catalytic activity that contributes to substrate binding and participates in nucleic acid metabolism under the DNA-PKcs regulation (164, 165). The clinical and immunological phenotype of patients showing Artemis deficiency largely depends on the type of variant in the *DCLRE1C* gene. While Artemis protein is involved in V(D)J antigen receptor gene rearrangement in T and B cell development, biallelic null variants in *DCLRE1C* cause T-B- SCID associated with increased cellular radiosensitivity (165, 166), and hypomorphic variants are linked to more complex genotype–phenotype correlations, with atypical combined immunodeficiency and immune dysregulation, Omenn syndrome, hyperimmunoglobulin M syndrome, and antibody deficiency (167–170).

Immune deficiency and immune dysregulation: The immunogenetic variability of Artemis deficiency is accompanied by a marked phenotypic heterogeneity ranging from relatively mild childhood sinopulmonary infections and an EBV-related lymphoproliferative disease to an early-onset BCG-itis, cerebral abscess, granulomatous skin lesions, warts, and intractable diarrhea (171). T-B-NK+ SCID with agammaglobulinemia was documented in a proportion of patients with variants in *DCLRE1C*, but CID with immune dysregulation presents with low RTE and naïve T cells, restricted T cell antigen receptor repertoire, defective T cell proliferative response to mitogens, and B cell deficiency with IgA, IgM, IgG, or IgG2 subclass deficiency (166, 169, 171, 172). Immune dysregulation in the form of autoimmune and inflammatory disorders, such as celiac disease, inflammatory bowel disease, juvenile idiopathic arthritis, vitiligo, Hashimoto thyroiditis, and autoimmune hemolytic anemia, thrombocytopenia, and neutropenia, was reported in patients affected with hypomorphic *DCLRE1C* variants (171).

Malignancy: Chromosomal instability and increased cellular hypersensitivity to radiation are the most plausible predisposing factors to malignant transformation in DNA DSB repair disorders. In patients with Artemis deficiency and SCID, as well as immunodeficiency due to hypomorphic variants in *DCLRE1C*, non-Hodgkin and Hodgkin lymphoma, localized in cervical and retriperitoneal lymph nodes, the liver, spleen, colon, lung, and adrenal gland, and acute lymphoblastic leukemia were noted in 17%–21% of the patients studied (171–173). Importantly, the average time of malignancy diagnosis in patients with hypomorphic *DCLRE1C* variants is 10 months only, and the development of cancer increases the mortality in Artemis deficiency by approximately twofold (171). Therefore, awareness of the malignancy risk and taking measures to achieve immune reconstitution with HSCT is of paramount importance. Lentiviral gene therapy for Artemis-deficient SCID is a promising perspective for the affected children, offering genetic and functional correction of T and B cells (174, 175).

## 4 NK cell deficiencies

### 4.1 Minichromosome maintenance complex member 4 deficiency

Molecular pathology: The *minichromosome maintenance 4* (*MCM4*) gene encodes an enzymatic protein which, together with MCM10 and Go-Ichi-Ni-San (GINS) 1 and 4, forms the CDC45-MCM-GINS (CMG) helicase complex (176, 177). The MCM4, MCM10, GINS1, and GINS4 complex-containing CMG helicase unwinds the DNA double helix and thereby enables access of polymerases to single-stranded DNA, thus playing an essential role in DNA replication. The MCM components are the catalytic activity center of the helicase, as each of them shows ATPase enzymatic function. As CMG helicase activity is required in any proliferating cell type, it raises the question of why NK cells are a particularly sensitive cell line to damaging variants in the *MCM4*, *MCM10*, *GINS1*, and *GINS4* genes. Consequently, replication stress, DNA damage, and cell cycle arrest result in impaired NK cell survival, differentiation, maturation, and function (176, 177). Along with NK cell deficiency, increased chromosomal breakage accompanied by short stature, failure to thrive, and microcephaly with adrenal glucocorticoid failure was demonstrated in children who were bearing homozygous splice site variants in MCM4 (178, 179).

Immune deficiency and immune dysregulation: Patients affected with MCM4 deficiency demonstrated a selective NK cell defect characterized by a lack of the CD56^dim^ subset. The loss of CD56^dim^ NK cells resulted from a specific defect in MCM4-dependent cell division and CD56bright NK cell proliferation and maturation. The phenotypic syndromic features, including microcephaly, growth retardation, and adrenal glucocorticoid failure, may reflect the ubiquitous but heterogeneous effects of the variants in the *MCM4* gene in various tissues (179). Due to the antitumor and antiviral cytotoxicity of NK cells, impairment of their differentiation and maturation predisposes the affected patients to viral infections, particularly to herpes viruses, including EBV (180, 181).

Malignancy: Important factors predisposing patients with variants in *MCM4* to developing cancer are both the genetic underpinnings and the consequent NK cell deficiency. While MCM4 is a component of the enzymatic protein of CMG helicase, whose activity is essential for DNA replication, dysfunctional MCM4 is associated with replication stress and DNA instability. Furthermore, the compromised NK cell cytotoxic immune response to oncogenic viruses, including EBV, is another risk factor for EBV-driven lymphomagenesis (179, 181).

### 4.2 Go-Ichi-Ni-San 1 deficiency

Molecular pathology: Go-Ichi-Ni-San (GINS) is a tetrameric polypeptide complex containing four chains, Sld5 (Go), Psf1 (Ichi), Psf2 (Ni), and Psf3 (San), which, along with Cdc45 and the heterohexameric MCM2-7, forms a CMG helicase. GINS is essential for DNA replication, mediating a large number of tightly regulated interactions with replication protein factors. The replicative CMG helicase unwinds DNA bidirectionally from the site of replication origin (182). Initiating DNA replication is a central two-step process, which involves loading of the MCM2–7 helicase onto the DNA strand and its activation by Cdc45 and GINS (183). Three models of GINS interactions with DNA have been hypothesized, depending on the association with a single- or double-stranded DNA or binding DNA exclusively during replication stress (184, 185). Compound heterozygous variants in *PSF1*, a.k.a. *GINS1* gene, were hitherto demonstrated in five patients who presented with intrauterine and postnatal growth retardation and mild facial dysmorphism (186).

Immune deficiency and immune dysregulation: Patients affected with compound heterozygous variants in *GINS1* suffered from recurrent viral and also some bacterial infections. They also presented with immune dysregulation autoimmune disorders, such as autoimmune hemolytic anemia, hypothyroidism, and enteropathy (186). The immune deficiency was characterized by an almost complete lack of circulating NK cells, both CD56^bright^ and CD56^dim^ subsets, and no increase in the proportion of NK cells after stimulation with IL-2 and IL-15. NK cell deficiency was accompanied with chronic neutropenia with a reduced bone marrow metamyelocyte compartment, which was restored by granulocyte colony-stimulating factor (G-CSF). This small cohort affected with GINS1 deficiency underwent a thorough immunological research, which evaluated the B and T cell compartment, T cell proliferative response to mitogens, and neutrophil oxidative burst test, the results of which were normal (186).

Malignancy: GINS1 deficiency is associated with impaired GINS complex assembly, replication stress, impaired checkpoint signaling, defective cell cycle control, and DNA instability. Taking into account all of these genomic phenomena and the replicative helicase-related NK cell deficiency, it may be assumed that they pose an increased risk of carcinogenesis. Thus far, one patient among the small cohort of GINS1 deficiency-affected individuals has developed osteosarcoma (186).

### 4.3 Go-Ichi-Ni-San 4 deficiency

Molecular pathology: Go-Ichi-Ni-San (GINS) complex subunit 4, a.k.a. Sld5, is one of four essential components of the CMG replicative helicase. Compound heterozygous pathogenic variants in the *GINS4* gene were demonstrated in two siblings who suffered from recurrent viral infections, with VZV, CMV, and HSV1, yet the susceptibility to infections and the clinical phenotype were variable among these affected individuals. One of them also manifested bacterial infectious diseases, such as pneumonia, sinusitis, otitis, dental abscess, and localized BCG, reflecting profound neutropenia and NK cell deficiency. Whereas this male sibling presented with syndromic features including an intrauterine growth restriction and postnatal growth delay, his sister had a normal physical development, and the course of infections she suffered from was significantly milder (187).

Immune deficiency and immune dysregulation: A novel disease-causing loss-of-function variant in GINS4 was associated with symptomatology resembling other syndromes occurring due to variants in genes encoding components of the CMG helicase. However, the genotype–phenotype relationships are complex, implicating further considerations regarding the roles of particular replicative CMG helicase proteins (187). GINS4 deficiency is linked to a cell cycle and proliferation delay, which may result from slower DNA replication and lead to DNA damage accumulation throughout the genome, interfering with cell development. The impaired NK cell maturation in GINS4 deficiency is admittedly detected at the stage of terminal differentiation, but the earliest hematopoietic precursors are actually affected, leading to changes in downstream lineage decisions (187).

Malignancy: In two GINS4-deficient siblings, malignancy was not demonstrated. However, due to a damaging impact upon the expression and assembly of the GINS complex with a delayed cell cycle progression and a causal link with impaired NK cell homeostasis and chronic viral infections, it may be assumed that the genomic underpinnings may pose an increased risk of carcinogenesis (181).

### 4.4 Minichromosome maintenance complex member 10 deficiency

Molecular pathology: Minichromosome maintenance complex member 10 (MCM10) is an essential replication factor unique to the replication machinery, as it promotes DNA unwinding and DNA synthesis. MCM10 lacks any enzymatic activity and any catalytic motifs. The ability to bind both single- and double-stranded DNA is vital for its function to activate CMG replicative helicase. During DNA elongation, MCM10 also interacts with tightly regulated replication protein factors, such as proliferating cell nuclear antigen (PCNA) and DNA polymerase α. An additional MCM10 role was demonstrated in protecting the genome integrity and engaging the DNA repair machinery to counteract the replication stress (188).

Immune deficiency and immune dysregulation: Compound heterozygous variants in the *MCM10* gene, causing a significant decrease in the amount of functional MCM10, manifested as clinically severe and fatal susceptibility to CMV herpes virus infection associated with hemophagocytic lymphohistiocytosis (HLH) (189). In this young pediatric patient, a lack of terminally mature CD56^bright^ NK cell deficiency was demonstrated, along with a slightly decreased B cell population and effector and memory T cell subsets. Inefficient DNA replication, impaired cell cycle progression, telomere loss, and default induction of DNA damage response pathways leading to genomic instability during NK cell differentiation may cause defective NK cell development (190).

Malignancy: MCM10 was identified as a suppressor of chromosomal breakage by counteracting the replication stress that contributes to tumorigenesis. It is likely that upregulated replication proteins as tumor cells increase their rate of proliferation, as it was observed in neuroblastoma or Ewing sarcoma, but homozygous and heterozygous deletions were also reported in prostate and lung cancers. Clinical awareness to identify patients with MCM10 deficiency is therefore required to estimate the malignancy risk (191).

## 5 Other immunodeficiencies

### 5.1 Polymerase ϵ subunit 2 deficiency

Molecular pathology: DNA polymerase ϵ (Pol ϵ) is a four-subunit complex belonging to the replisome, a large DNA replication origin depending on cell type and developmental stage. Polymerases δ and ϵ, due to their catalytic role, display a replicative function for the lagging and leading DNA strands, respectively. The high fidelity of DNA replication is based on a three-step process, including base selection, exonucleolytic proofreading, and DNA mismatch repair. Spontaneous variants due to replication errors caused by nucleotide misincorporation are corrected by Pol δ and Pol ϵ, which possess functional exonuclease domains (192). Variants affecting the catalytic domain of the *POLE* gene lead to impairments in the structure of catalytic Pol ϵ core and the CMG helicase complex, and the catalytic Pol subunits represent most cancer-associated variants. The important causative factor of malignant transformation is also the low fidelity of DNA replication associated with altered proofreading in the exonuclease domain due to germline pathogenic variants in *POLE*, predisposing to adenomatous polyps and colorectal and endometrial cancers (192, 193).

Immune deficiency and immune dysregulation: A homozygous splice-site variant in the *POLE2* gene in one pediatric patient with combined immunodeficiency was hitherto demonstrated. The boy presented with omphalitis and erythroderma in the neonatal period, systemic BCG infection as an adverse effect following immunization (AEFI), recurrent respiratory infections, lymphadenopathy, hepatopathy, and autoimmune complications, such as hypothyroidism and diabetes (194). The immunological workup revealed agammaglobulinemia, absence of B lymphocytes, T cell lymphopenia, undetectable T cell receptor excision circles (TRECs), increased proportion of T effector memory cells, reduced number of NK cells with accumulation of CD56^bright^ cells, and neutropenia (194). Whereas this is the first report of a patient with a germline variant in *POLE2* and profound combined immunodeficiency with an early block in lymphocyte development, it may be assumed that defective DNA replication is the causative factor leading to defective lymphocyte differentiation and maturation.

Malignancy: In this patient, the development of malignancy was not reported, and neither his siblings nor his parents, who were not homozygous for the variant, were affected with cancer. Further observation of the homozygous proband and heterozygous family members and special awareness are required, as the risk of tumorigenesis in the reported variant may be increased due to genomic instability, asynchronous cell cycle progression, and profound immunodeficiency (194).

### 5.2 Rothmund–Thomson syndrome

Molecular pathology: Rothmund–Thomson syndrome (RTS) is an autosomal recessive multisystemic disorder characterized by a highly variable phenotype and genetic heterogeneity associated with the diversity of the risk of carcinogenesis. Biallelic pathogenic variants in the *RecQ like helicase 4* (*RECQL4*) and *anaphase-promoting complex subunit 1* (*ANAPC1*) genes most frequently account for RTS, and *DNA2* and *cysteine-rich interactor of PDZ three* (*CRIPT*) genes were also implicated in a minority of affected patients (195). Referring to clinical phenotypes along with molecular genetic underpinnings, RTS may be categorized as type 1 and type 2. RTS1 results from pathogenic variants in *ANAPC1* and manifests with poikiloderma, ectodermal dysplasia, and juvenile cataracts, while RTS2, related to pathogenic variants in *RECQL4*, is characterized by poikiloderma, bone anomalies, and an increased risk of skin cancer and osteosarcoma later in life (195, 196). Missense variants in *RECQL4* causing RTS2 are rare, whereas frameshift, nonsense, and splice variants predominate (196). Moreover, besides RTS, the *RECQL4* gene has been implicated in the pathogenesis of other syndromic disorders, such as Baller–Gerold syndrome with poikiloderma, craniosynostosis, and skeletal abnormalities or RAPADILINO (*ra*dial ray malformations, *pa*tellar hypoplasia and cleft palate, *di*arrhea and dislocated joints, *li*ttle size and limb malformation, *no*se slender and normal intelligence) (197).

RECQL4 is an ATP-dependent DNA helicase maintaining genome stability and participating in the initiation of DNA replication through its N-terminus, which recruits essential replication factors to the origin of replication and DNA synthesis. The RECQL4 helicase SLD2 domain forms cell-cycle-dependent, chromatin-bound protein complexes containing core replisome factors MCM10, MCM2-7, CDC45, and GINS at the replication origins. Furthermore, RECQL4 localizes not only in the nucleus but also in the cytoplasm and plays a role in mitochondrial DNA synthesis (198). Upon the interaction with MRE11–RAD50–NBS1, which senses DNA DSB, the RECQL4 helicase, playing a role in DNA end resection, is thereby involved in homologous recombination-dependent DNA DSB repair (199).

Immune deficiency and immune dysregulation: Immune deficiency was admittedly not included into the classical symptomatology of Rothmund–Thomson syndrome due to variants causing RECQL4 helicase dysfunction. In one pediatric patient affected with RTS, with cutaneous granulomatosis complicating a primary *Varicella zoster* virus infection, immunodeficiency was reported (200). The girl presented with low serum IgM, IgG2 subclass, specific antibodies against polio, measles, and hepatitis B viruses, as well as pneumococcal antigens and isohemagglutinins, and also a decreased percentage of switched memory B cells and NK cells. It was also hypothesized that, due to the predominant RELCQ4 helicase function in DNA replication, it may play a role as a regulator of hematopoiesis. Therefore, somatic variants in the *RELCQ4* gene may affect the leukocyte development and increase DNA damage and lymphocyte apoptosis (201).

Malignancy: Whereas RECQL4 helicase is implicated in maintaining genomic integrity, DNA replication, double-strand break repair, and mitochondrial DNA biogenesis, genomic instability caused by pathogenic variants in *RECQL4* is associated with predisposition to malignancy. It poses a particular risk to developing osteosarcoma and skin cancer, as well as leukemia (202, 203), and truncating variants in *RECQL4* are linked to the heightened predisposition to osteosarcoma (195). Due to the rarity of RTS, heterogeneity of clinical manifestations, and complex phenotype–geneotype relationships, high vigilance of the syndrome, an early diagnosis, and thorough monitoring of affected patients are essential.

The biological mechanisms relevant for the pathogenesis of DNA instability in inborn errors of immunity are summarized in **Table 2**.

**Table 2:** Biological mechanisms of DNA instability in inborn errors of immunity.

DNA repair pathway			
Non-homologous end joining defects	Chromosomal instability syndromes	DNA replication defects	Mismatch repair disorders
Artemis deficiency DNA Ligase IV deficiency Cernunnos/XLF deficiency DNA-PKcs deficiency	Ataxia–telangiectasia Nijmegen breakage syndrome Bloom syndrome RNF168/RIDDLIN deficiency ICF syndrome	DNA ligase I deficiency NSMCE3 deficiency POLE deficiency MCM4 deficiency MCM10 deficiency GINS1 deficiency GINS4 deficiency Rothmund–Thomson syndrome	PMS2 deficiency/CMMRD

**Figure 2:**
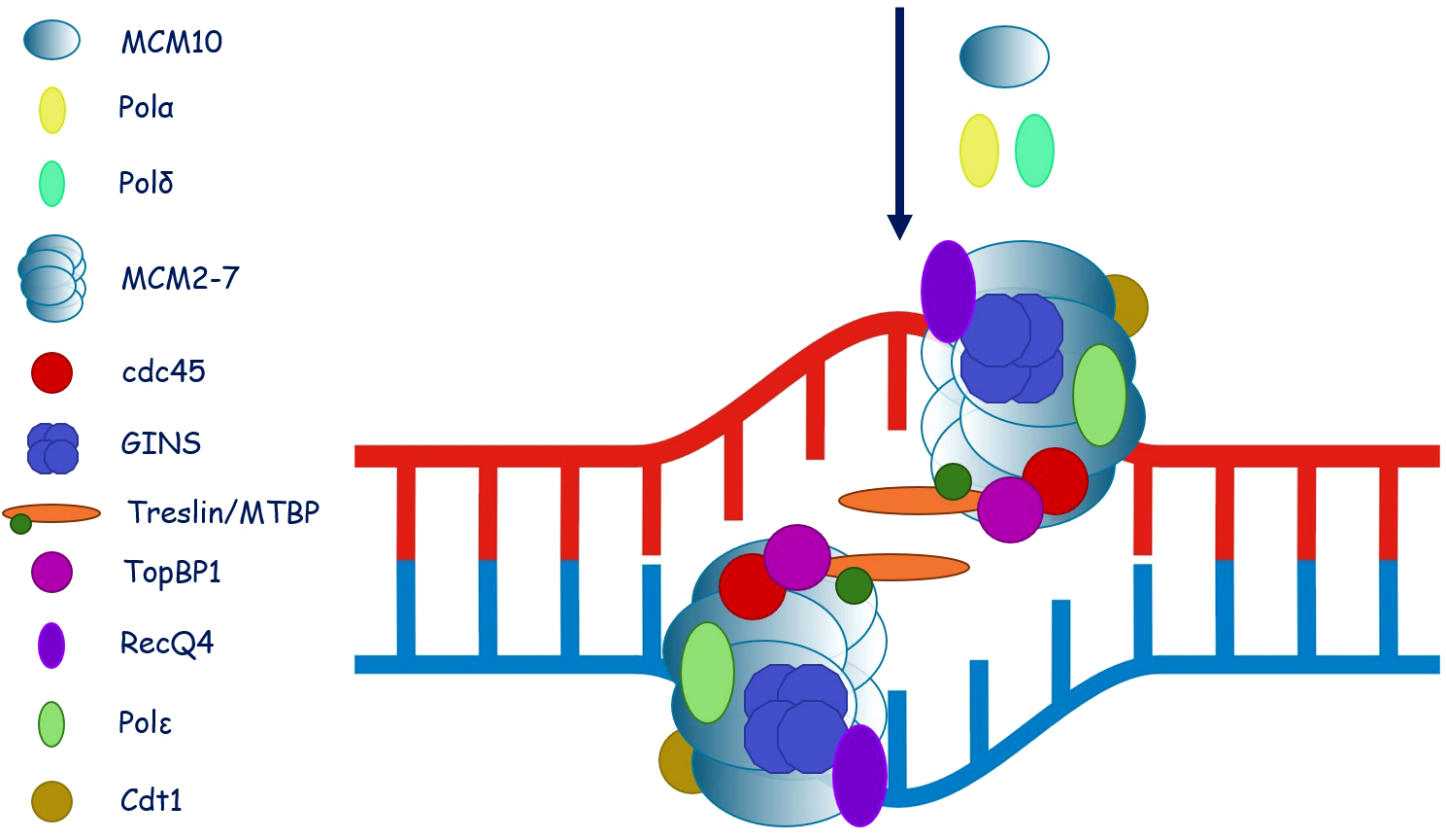
DNA replication process with a protein complex involved in the replication initiation [according to (185)].

The DNA replication pathway with engagement of specific protein biomarkers, relevant as genome guardians and playing a role in immune cell development and function, is displayed in **Figure 2**.

## 6 Therapeutic approach

DNA instability disorders require multidisciplinary surveillance and monitoring, as several cancers occur at a younger age and higher prevalence than in the general population. Aberrant DNA repair and oxidative stress make these patients not only vulnerable to malignant transformation but also they present with remarkable susceptibility to anticancer treatments, radiotherapy and chemotherapy. In particular, alkylating agents, exerting their anticancer effect through DNA damage, show a prominent toxicity, and reduced-intensity regimens are indicated (41). Due to the high cancer risk, predisposition to severe infections, autoimmunity, and autoinflammation, allogeneic (allo) HSCT has been, for many years, the only definitive, curative therapeutic option; however, this requires the availability of a suitable donor and is associated with immunological complications, such as graft-versus-host disease (GVHD), graft failure, secondary malignancy, immunosuppression, endocrinopathy, and growth restriction (175).

Using the host’s autologous cells undergoing gene transduction, this method abrogates the need for donor availability and post-allo-HSCT complications. It enables the correction of genetic disorders caused by loss-of-function variants by gene modification using autologous CD34+ cells transfected with lentiviral vectors (175, 204). Loss-of-function variants in *DCLRE1C* encoding Artemis lead to T-B-NK+ SCID with compromised DSB repair and radiosensitivity, which is poorly responsive to allo-HSCT. The procedure of administration of autologous CD34+ bone marrow cells transduced with a lentiviral construct containing *DCLRE1C* DNA driven by an autologous promoter showed biosafety and led to immune reconstitution in a group of Artemis-deficient SCID infants (174). Therefore, further studies are warranted regarding the safety of pharmacological conditioning with low-exposure busulfan, the effectiveness of the procedure as immune reconstitution, long-term monitoring for complications, and finally, to propose other genomic instability disorders as candidates for gene therapy.
